# Factors associated with self-reported work ability among people with multiple sclerosis in Sweden

**DOI:** 10.1177/20552173241304324

**Published:** 2025-01-07

**Authors:** Fitsum Sebsibe Teni, Alejandra Machado, Jessica Dervish, Katharina Fink, Hanna Gyllensten, Emilie Friberg

**Affiliations:** Division of Insurance Medicine, Department of Clinical Neuroscience, Karolinska Institutet, Stockholm, Sweden; Division of Neurology, Department of Clinical Neuroscience, 27106 Karolinska Institutet, Stockholm, Sweden; Institute of Health and Care Sciences, Sahlgrenska Academy, 3570 University of Gothenburg, Gothenburg, Sweden; Division of Insurance Medicine, Department of Clinical Neuroscience, 27106 Karolinska Institutet, Stockholm, Sweden

**Keywords:** Expanded disability status scale, EQ-5D, fatigue, work ability index, work ability score

## Abstract

**Background:**

Work ability index (WAI) is an instrument that measures work ability in workplace surveys and health examinations in occupational health and research. It has been used in different population groups. But research is limited among people with multiple sclerosis (PwMS).

**Objective:**

To determine the factors associated with work ability among PwMS in Sweden.

**Methods:**

A total of 4103 PwMS who answered a web-based survey were included in the analysis. Work ability was assessed using the work ability score (WAS) component of WAI. Univariable and multivariable linear regression analyses were performed to assess the association of sociodemographic, clinical, and self-reported health variables with WAS.

**Results:**

Just over half of the PwMS reported *good* (37.0%) or *excellent* (16.3%) WAS. The overall mean WAS was 6.9 (standard deviation = 2.8). Health-related quality of life (R-squared = 31.6%), fatigue (28.3%), occupation (22.6%), and expanded disability status scale (EDSS) score (18.1%), explained the highest proportions of variation in WAS, individually. In the adjusted model, occupation, EDSS score, and fatigue had the strongest associations with WAS with significantly lower scores in those with no occupation, higher EDSS score, and severe fatigue levels.

**Conclusion:**

Work ability among PwMS was lower than in the general population in Sweden. Occupation, EDSS score and fatigue were among the most important factors associated with work ability.

## Introduction

Work-related outcomes such as unemployment, absenteeism, and presenteeism among people with multiple sclerosis (PwMS) have been linked with important clinical and self-reported health variables. Of these, fatigue has been associated with not having paid work, higher levels of absenteeism, reduced work hours, presenteeism, and lower work ability.^1^ Early job loss, early retirement, and unemployment have also been associated with higher expanded disability status scale (EDSS) score.^2,3^ The type of MS has also been linked to the impact MS has on work-related outcomes. An American study showed lower fulltime employment and higher levels of disability before turning 60 among PwMS with progressive MS than those with the relapsing course.^4^

Work ability has been described as the resulting balance between demands of work and an individual's resources such as health, competence, and education among others.^5^ Work ability index (WAI) is a questionnaire widely used to assess work ability in clinical and research context. A summarized index is calculated using the questions which address the workers’ work demands, health status, and resources of the worker. Among the seven items is the assessment of one's current work ability in comparison to life time best (work ability score, WAS). The questionnaire is translated into many languages and its validity and reliability have been assessed in different countries,^6^ including in Sweden where its usability and predictive ability have been studied.^7,8^

The WAI and its WAS component have associations with other measures of work, most notably WAI and WAS with risk of sickness absence.^7,9–11^ They have also predicted the risk of disability pension among workers with lower work ability.^12^ Furthermore, WAI has been linked to employment status^13–15^ and work productivity.^16^ Consequently, studies on WAI/WAS could provide useful insights on the risk of other work outcomes.

In MS research, few studies have employed WAI/WAS.^1,3,17,18^ These have been conducted in the Netherlands on capability set for work^17^ and the use of WAS as part of assessing self-reported occupational functioning^1,18^ and in France on social and professional impact of MS.^3^ However, WAI/WAS has not been not studied as the main outcome measure. Furthermore, to the best of our knowledge, there are no studies on WAI/WAS and the associated factors among PwMS in Sweden. Hence, the objective of the present study was to determine the factors associated with work ability among PwMS in Sweden.

## Methodology

### Study design

The present study used information from a web-based cross-sectional survey conducted in 2021 on the health and work life of PwMS in Sweden.

### Study population

The survey was sent to all PwMS aged between 20 and 51 years included in the Swedish MS registry, the age range was determined by the focus of the survey which revolved around strategies and choices in relation to work and life situation (i.e., family).^19^ Of the total 8458 individuals invited to participate (with four reminders), 4412 (52%) responded to the survey. Overall, nonrespondents constituted younger, men, and those with lower income or born outside Sweden. After exclusion for missing observations in the WAS, disease-modifying therapy (DMT) use, type of MS, fatigue, education, presence of another long-term illness sequentially, 4103 PwMS were included in the analysis.

### Data

Data on work ability was obtained from the survey. On the WAS component of WAI, respondents rated their current work ability in comparison to their life time best from 0 (“cannot work at all”) to a maximum of 10 (“work ability at its best”).^6^ The WAS of each PwMS was categorized as *poor* (0–5), *moderate* (6–7), *good* (8–9), and *excellent* (10).^20^ Studies demonstrated the association between the single WAS question and the WAI score based on all items indicating it could be employed independently in situations where the full WAI may not be feasible.^11,20^ In the survey data on the WAS component of WAI was collected. Accordingly, PwMS were asked “*How is your current work ability in comparison to the best it has been?*”.

The type of occupation among the PwMS was assessed with an open-ended question. The answers were categorized into “managerial,” “office,” and “manual” occupation on the basis of the physical demand related to it (details in the supplementary material).

Data on the presence of long-term disease or disability other than MS (hereafter long-term disease besides MS) was also collected in the survey. Treatment with DMT among PwMS, in two years before the survey, was categorized as high-efficacy, non-high-efficacy, and no DMTs, as performed in a recent study^21^ (details in the Supplemental material).

Fatigue was assessed through self-reporting, using the NeuroQol fatigue short form. The scores were categorized into milder (<50) or more severe (>50) fatigue using the mean in the reference population (50) as a cutoff.

Data on health-related quality of life was collected using the EuroQol health state questionnaire with five dimensions (EQ-5D), the EQ-5D-3L version.

The data collected in the survey was linked to sociodemographic data (education and type of living area) from Statistics Sweden's Longitudinal Integrated Database for Health Insurance and Labor Market Studies (LISA).^22^ In addition, data on sex, age, the type of MS, date of MS onset, diagnosis and treatment start times, status of pediatric onset MS (<18 years), EDSS score as well as DMT use were obtained from the Swedish MS registry.

### Statistical analysis

Descriptive statistics, through proportions and means, were used to present sociodemographic and clinical characteristics of PwMS. Analysis of WAS by specific sociodemographic (occupation), clinical (EDSS score categories), and self-reported health (fatigue T-score categories) variables was also performed.

Crude/unadjusted linear regression analyses were conducted to assess the association between several individual sociodemographic (age, sex, education, and occupation) and clinical (type of MS, EDSS score, long-term disease besides MS) and self-reported health (fatigue and health-related quality of life) variables and WAS. Furthermore, adjusted linear regression analyses were performed in a stepwise manner by entering blocks of sociodemographic (sex, age education, and living area), clinical (EDSS score, presence of long-term illness, DMT use, time since treatment start), and self-reported health (fatigue T score) variables. The variable health-related quality of life was not included in the adjusted model considering the similarity it has, in construct and the high correlation (spearman's rank correlation = 0.71), with the outcome variable, WAS. Interaction effects between EDSS score and fatigue as well as EDSS score and DMT use were assessed in the adjusted model. Furthermore, subgroup analyses with regression models in each of EDSS score 0, 1–2.5, 3–5.5, and 6–9.5 were performed.

Normality of the distribution of WAS was assessed using skewness (−1.06) and kurtosis (3.3). As heteroscedasticity was found based on Breusch-Pagan test, robust standard errors were reported for the estimates in all the regression models.^23^ P-value of 0.05 was used as a cutoff for statistical significance and analyses were conducted using R version 4.1.2.^24^

## Results

### Demographic and clinical characteristics

Of the 4103 PwMS in the study 71.1% were women. The overall mean age was 40.3 years with nearly half (44.0%) of the PwMS between 36 and 45 years. Nearly two-thirds (61.4%) of the PwMS attended university level education and 69.2% had office jobs (Table 1).

**Table 1.** Demographic and clinical characteristics of people with MS.

Variable		Total n (%)
**Total**		4103 (100)
**Sex**	Women	2919 (71.1)
	Men	1184 (28.9)
**Age group**	20–25	121 (2.9)
	26–35	966 (23.5)
	36–45	1804 (44.0)
	46–51	1212 (29.5)
**Age (mean (SD))**		40.3 (7.2)
**Age (median (IQR))**		41 (11)
**Education**	Up to high school	1584 (38.6)
	University/college	2519 (61.4)
**Occupation**	Managerial	458 (11.2)
	Office	2839 (69.2)
	Manual	277 (6.8)
	No occupation	529 (12.9)
**Type of living area**	Big cities	1856 (45.2)
	Medium-sized cities	1557 (37.9)
	Rural areas	690 (16.8)
**Type of MS**	Relapsing-remitting	3828 (93.3)
	Primary progressive	66 (1.6)
	Secondary progressive	209 (5.1)
**Pediatric onset MS**	Yes	215 (5.2)
	No	3636 (88.6)
	Missing	252 (6.1)
**Time since MS onset in years (mean (SD))**		11.4 (7.2)
**Time since MS diagnosis in years (mean (SD))**		9.3 (6.3)
**Time since first MS treatment in years (mean (SD))**		8.6 (5.7)
**EDSS score (latest in the past 3 years)**	0–2.5	2691 (65.6)
	3–5.5	493 (12.0)
	6–9.5	145 (3.5)
	Missing	774 (18.9)
**Long-term disease or disability besides multiple sclerosis**	Yes	1265 (30.8)
	No	2838 (69.2)
**Fatigue T score (mean (SD))**		50.4 (9.9)
**Health-related quality of life (EQ-5D-3L) (mean (SD))**		0.86 (0.13)
**DMT use (latest in the last 2 years)**	Non-high efficacy	1167 (28.4)
	High-efficacy	2775 (67.6)
	No DMT	161 (3.9)

DMT: disease-modifying therapy; EDSS: expanded disability status scale; EQ-5D-3L: The three-level severity EuroQol five-dimension questionnaire; HE: high efficacy; IQR: interquartile range; MS: multiple sclerosis; SD: standard deviation.

Almost all (93.3%) of the PwMS had relapsing-remitting type of MS and just over 5% had a pediatric onset MS. The average time since MS onset was 11.4 years. Among the majority (65.6%) of the PwMS, the latest EDSS score in the past three years was within 0 to 2.5. About a third (30.8%) of the PwMS reported having another long-term disease besides MS. The mean overall fatigue T score was 50.4. The average health-related quality of life (EQ-5D) among the PwMS was 0.86. Around two-thirds (67.6%) and more than a quarter (28.4%) of the PwMS were on high-efficacy and non-high-efficacy DMTs, respectively (Table 1).

### Work ability score

Just over half of the PwMS reported either *good* (37.0%) or *excellent* (16.3%) WAS. The remaining reported *poor* (24.0%) or *moderate* (22.8%) levels of WAS. The overall mean WAS was 6.9 (SD = 2.8) with a median score of 8.0 (25th–75th percentile = 6.0–9.0).

### Work ability score by occupation

**Figure 1.**
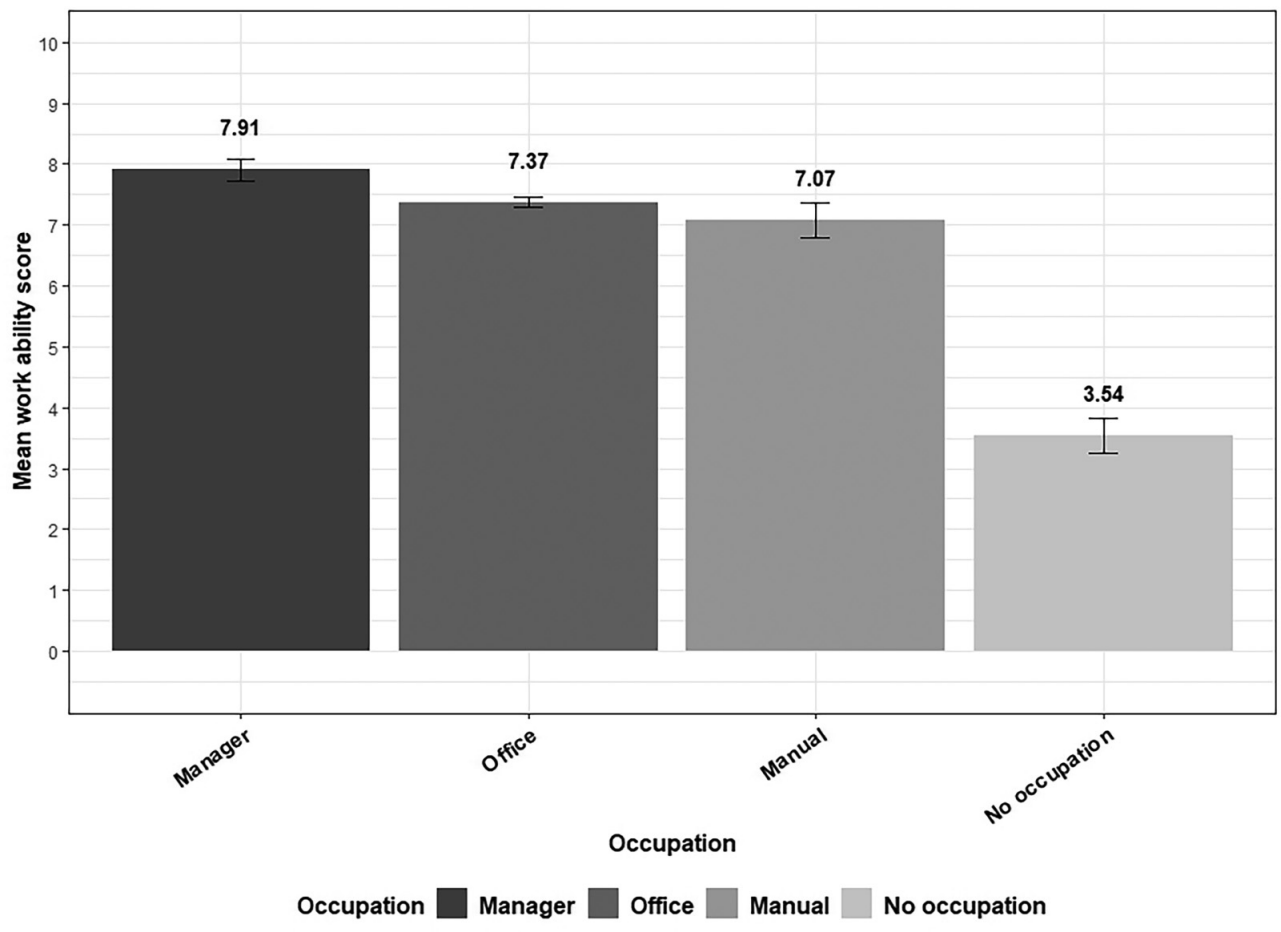
Work ability score by occupation among people with multiple sclerosis.

Across occupational groups, WAS showed that, on average, managers reported (mean = 7.9, standard deviation (SD) = 1.9) statistically significantly higher score than workers with office jobs (mean = 7.4, SD = 2.3), manual jobs (mean = 7.1, SD = 1.9), and those with no occupation (mean = 3.5, SD = 3.4; Figure 1).

### Work ability across disability levels

**Figure 2.**
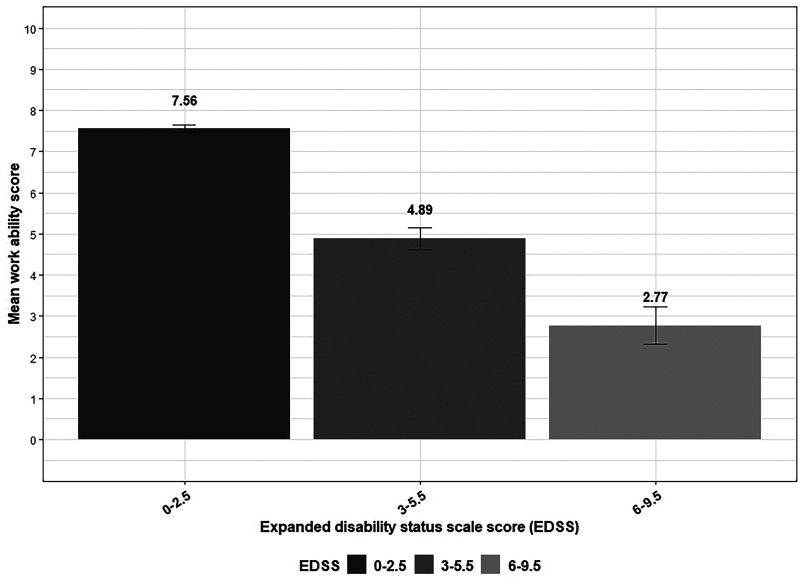
Work ability score by expanded disability status scale score categories among people with multiple sclerosis.

Overall, PwMS in the EDSS score category of 0–2.5 (lowest levels of disability) reported the highest mean WAS (mean = 7.6, SD = 2.3), while PwMS in the EDSS score category 6–9.5 reported the lowest (mean = 2.8, SD = 2.8; Figure 2).

### Work ability score by fatigue T score

The level of mean WAS by fatigue status showed that PwMS with fatigue T score of lower than 50 reported significantly higher mean WAS (mean = 8.5, SD = 1.6) than those who reported more severe levels of fatigue (mean = 5.6, SD = 2.8).

### Factors associated with work ability score among PwMS

Univariable linear regression analyses showed that all demographic and clinical variables except pediatric MS onset status had statistically significant associations with WAS. However, the variables age, sex, type of living area and time since treatment start, predicted WAS to a very small extent with explained variance of <1% (Table 2).

**Table 2.** Unadjusted linear regression models of the association of sociodemographic, clinical, and self-reported health variables with work ability score.

Variable	Unadjusted models			
	Estimate	RSE	P-value	R square (%)
Sex				0.17
Women (intercept)	6.84	0.05	**<0**.**0001**	
Men	0.25	0.09	**0**.**0063**	
Age group (years)				0.69
26–35 (intercept)	7.21	0.08	**<0**.**0001**	
20–25	0.15	0.25	0.5398	
36–45	−0.28	0.10	**0**.**0069**	
46–51	−0.59	0.12	**<0**.**0001**	
Education				3.55
Primary/secondary education (intercept)	6.26	0.08	**<0**.**0001**	
University education	1.07	0.09	**<0**.**0001**	
Occupation				22.59
Office job (intercept)	7.37	0.04	**<0**.**0001**	
Managerial	0.53	0.10	**<0**.**0001**	
Manual	−0.30	0.15	**0**.**0498**	
No occupation	−3.83	0.15	**<0**.**0001**	
Pediatric onset MS				0.0002
No (intercept)	6.93	0.05	**<0**.**0001**	
Yes	−0.02	0.21	0.9399	
Type of living area				0.52
Big cities (intercept)	7.13	0.06	**<0**.**0001**	
Small town/ suburb	−0.33	0.09	**0**.**0006**	
Sparsely populated area	−0.50	0.13	**<0**.**0001**	
Time from treatment start				0.52
Intercept	7.21	0.08	**<0**.**0001**	
Time from treatment start (years)	−0.03	0.01	**<0**.**0001**	
EDSS score				18.08
0–2.5 (intercept)	7.56	0.04	**<0**.**0001**	
3–5.5	−2.67	0.14	**<0**.**0001**	
6–9.5	−4.79	0.24	**<0**.**0001**	
Type of MS				9.06
Relapsing-remitting MS (intercept)	7.14	0.04	**<0**.**0001**	
Primary progressive	−2.65	0.36	**<0**.**0001**	
Secondary progressive	−3.51	0.22	**<0**.**0001**	
Fatigue T score				28.31
Lower than 50 (intercept)	8.53	0.04	**<0**.**0001**	
Higher than 50	−2.95	0.07	**<0**.**0001**	
Long-term disease or disability besides MS				5.15
No (intercept)	7.34	0.05	**<0**.**0001**	
Yes	−1.36	0.10	**<0**.**0001**	
DMTs use				1.24
Non-HE DMTs (intercept)	7.40	0.07	**<0**.**0001**	
HE DMTs	−0.65	0.09	**<0**.**0001**	
No DMTs	−0.94	0.28	**0**.**0008**	
Health-related quality of life (EQ-5D)				31.59
Lower than median (<0.9028) (intercept)	5.36	0.06	**<0**.**0001**	
Equal to or higher than median (≥0.9028)	3.10	0.07	**<0**.**0001**	

P-value <0.05 are shown in bold.

DMT: disease-modifying therapy; EDSS: expanded disability status scale; EQ-5D: EuroQol five-dimension questionnaire; HE: high efficacy; RSE: robust standard error; MS: multiple sclerosis.

Among the sociodemographic variables, occupation explained a large percentage (22.6%) of the variance in WAS. Accordingly, PwMS with managerial occupations reported significantly higher WAS than those with office jobs while individuals with manual occupations and those not working reported lower WAS (Table 2).

Regarding clinical factors, PwMS in the higher EDSS score categories had lower WAS than those in the 0 to 2.5 category. Individuals with progressive forms of MS also had significantly lower WAS compared to those with the relapsing type (Table 2).

In the unadjusted model, PwMS on high-efficacy or no DMTs reported significantly lower WAS than those on non-high-efficacy DMTs. However, DMT use explained just above 1% of the variation in WAS.

PwMS with more severe fatigue had almost three points lower WAS than those with milder fatigue. On the other hand, PwMS with higher health-related quality of life had more than three points higher score of WAS than those with lower health-related quality of life (Table 2).

In the adjusted regression models, the variance in WAS explained by the demographic, clinical, and self-reported health variables (fatigue), reached more than 51% in the final model (model 4). In the final adjusted model, WAS showed no significant difference between women and men. However, while older ages were associated with lower WAS, those who attended university education reported significantly higher WAS. In terms of occupation, PwMS with no occupation had significantly lower WAS. The statistically significant higher WAS in models 2 and 3 was, however, no longer significant in the final model (Table 3).

**Table 3.** Linear regression analysis on predictors of work ability score adjusted sequentially for demographic, clinical, and self-reported health variables.

Variable	Model 1			Model 2			Model 3			Model 4		
	Estimate	RSE	P-value	Estimate	RSE	P-value	Estimate	RSE	P-value	Estimate	RSE	P-value
Intercept	7.14	0.09	**<0**.**0001**	7.31	0.11	**<0**.**0001**	7.92	0.12	**<0**.**0001**	9.07	0.11	**<0**.**0001**
Sex												
Men	0.26	0.09	**0**.**0048**	0.36	0.08	**<0**.**0001**	0.31	0.08	**<0**.**0001**	0.05	0.07	0.4855
Age group												
20–25	0.15	0.25	0.5393	1.03	0.26	**<0**.**0001**	0.86	0.23	**0**.**0001**	0.80	0.20	**<0**.**0001**
36–45	−0.28	0.11	**0**.**0067**	−0.48	0.09	**<0**.**0001**	−0.39	0.09	**<0**.**0001**	−0.35	0.08	**<0**.**0001**
46–51	−0.60	0.12	**<0**.**0001**	−0.66	0.10	**<0**.**0001**	−0.49	0.10	**<0**.**0001**	−0.50	0.09	**<0**.**0001**
Education												
University education				0.65	0.09	**<0**.**0001**	0.58	0.08	**<0**.**0001**	0.37	0.07	**0**.**0001**
Occupation												
Managerial				0.54	0.10	**<0**.**0001**	0.34	0.10	**0**.**0004**	0.16	0.08	0.0544
Manual				−0.15	0.16	0.3494	−0.18	0.15	0.2166	−0.04	0.13	0.7451
No occupation				−3.73	0.15	**<0**.**0001**	−2.92	0.14	**<0**.**0001**	−2.61	0.13	**<0**.**0001**
Type of living area												
Small town/suburb				−0.14	0.08	0.0867						
Sparsely populated area				−0.21	0.11	0.0538						
EDSS score												
3–5.5							−1.89	0.13	**<0**.**0001**	−1.45	0.11	**<0**.**0001**
6–9.5							−2.65	0.25	**<0**.**0001**	−2.25	0.23	**<0**.**0001**
Type of MS												
Primary progressive							−0.82	0.29	0.6170	−0.55	0.29	0.0569
Secondary progressive							−1.13	0.21	**0**.**0046**	−0.87	0.19	**<0**.**0001**
Long-term illness/disability besides MS												
Yes							−1.01	0.08	**<0**.**0001**	−0.59	0.07	**<0**.**0001**
DMT use												
High-efficacy DMTs							−0.30	0.08	**<0**.**0001**	−0.20	0.07	**0**.**0022**
No DMTs							0.01	0.20	0.9648	−0.05	0.18	0.7958
Time from treatment start							0.02	0.01	**0**.**0012**	0.01	0.01	0.0792
Fatigue T score												
Higher than 50										−2.17	0.06	**<0**.**0001**
R^2^ / adjusted R^2^ (%)	0.78			25.43			37.99			51.43		

Reference group: women; 26–35 years; primary/secondary education; office job, big cities; EDSS score: 0–2.5; relapsing-remitting MS; fatigue T score lower than 50; no long-term disease or disability besides MS; non-high efficacy DMTs.

P-values <0.05 are shown in bold.

DMT: disease-modifying therapy; EDSS: expanded disability status scale; RSE: robust standard error; MS: multiple sclerosis.

The clinical variables largely remained statistically significant predictors of WAS in the final model. Higher EDSS score categories predicted significantly lower WAS, with the lowest WAS observed among PwMS in the 6 to 9.5 category. Furthermore, PwMS with long-term disease besides MS reported significantly lower WAS than those without one. While primary progressive MS was not significantly associated with WAS, individuals with secondary progressive MS had lower WAS than those with relapsing-remitting type. In both model 3 and the final model (model 4), WAS among PwMS on high-efficacy DMTs remained significantly lower than those on non-high-efficacy DMTs (Table 3).

The self-reported health variable fatigue also remained significantly associated with WAS in the final adjusted model. However, the strength of the association was attenuated (Table 3). Results of the interaction effects and subgroup analyses are summarized in the Supplemental material section.

## Discussion

The present study was based on data of PwMS responding to the survey with a response rate of 52% which is considered very good in the context of the usually lower response rates among surveys.^25^ Just over half of the PwMS in the study reported *good* to e*xcellent* WAS levels. The WAS showed a gradient with disability levels with better work ability noted among PwMS with milder disability. Although most of the variables had significant associations with WAS, occupational status, EDSS score, and fatigue explained large proportions of variance in WAS.

The mean and median WAS of PwMS in the present study were comparable to findings of studies among people with relapsing-remitting MS in the Netherlands.^1,17^ The PwMS in the two studies had similar median age and EDSS score but higher proportions of women (77.3% and 78.0%) than the present study (71.1%).^1,17^ In comparison to another study from the Netherlands, our finding showed a slightly higher (median, 8.0 vs. 7.0) WAS which could partly be due to the slightly higher median EDSS score of 2 in the cited study (vs. 1.5 in ours).^18^ In comparison to the general population in Sweden (WAS = 8.25) with a comparable mean age, we found a lower mean WAS among PwMS in the present study.^7^

Occupation explained a large proportion of the variance in WAS in the unadjusted model where PwMS with managerial job reported higher WAS than office workers while those with manual jobs or no occupation reported lower WAS than office workers. This aligns with prior studies where physically demanding jobs were associated with lower WAS.^26–28^ The association remained in the final adjusted model for those with no occupation. The lower WAS among PwMS with no occupation could partly be related to possible long-term impact of MS restricting the ability to return to full work capacity. Difficulty to readapt to work after long-term absence could also have contributed to this. Similarly, in a previous study unemployed PwMS reported more comorbidities, higher EDSS score, and depressive symptoms among others.^29^ This indicates that important clinical variables including comorbidities and EDSS score could partly influence occupational status, as shown previously.^2,30^ These variables were highly associated with WAS in our study.

Among clinical variables, people with secondary progressive MS reported lower WAS than those with relapsing-remitting MS. Previous studies also showed higher levels of unemployment among individuals with progressive MS.^31^ Similarly, worse health, lower treatment use and more hospitalization have been reported among people with secondary progressive MS than with relapsing-remitting type.^32^ The presence of a long-term disease besides MS was also associated with lower WAS and remained significant in the final model. The association of comorbidities with WAS has also been shown previously.^33^

Disability-EDSS score-explained a large proportion of the variance in WAS (18.1%) in the unadjusted model and remained significant after adjustments, with higher EDSS score predicting lower WAS. Hence, WAS showing gradient across EDSS score categories. However, in the study from the Netherlands, EDSS was not a significant predictor of WAS.^1^ Among the possible reasons for the difference could be studying only PwMS with relapsing-remitting MS, inclusion criteria of employment and exclusion based on a number of comorbidities in that study.^1^ The lower sample size (n = 173) in the cited study could also explain the difference with the present study.^1^

PwMS in the non-high-efficacy DMT use category reported significantly higher mean WAS than those on high-efficacy DMTs both in the unadjusted and adjusted linear regression models. The difference with PwMS on high-efficacy DMTs could partly reflect treatment choice for those with more active MS observed in the lower WAS. However, DMT use in itself explained only around 1% of the variation in WAS. Furthermore, the association was attenuated after accounting for demographic, clinical and self-reported health variables which could correspond with the explanation above that clinical course and severity of the disease (as shown in the discussion of the role of MS type (nearly three-quarters of people with SPMS were on high-efficacy DMTs) and EDSS score likely had influenced the type of DMT used. Similarly, in a previous study we found relatively fewer and more stable trends of sickness absence and/or disability pension days among PwMS on long-term non-high-efficacy DMTs than those escalating to high-efficacy DMTs which was also linked to more severe disease among the latter.^21^

Fatigue was highly associated with WAS explaining a large proportion of the variance in the unadjusted model and in the final model. This is in line with studies showing fatigue is one of the most common symptoms among PwMS and its debilitating effects are associated with unemployment, lower working hours, and increased sickness absence.^28,29,34^ The study from the Netherlands also showed comparable findings to ours where fatigue was associated with lower WAS.^1^ Among the possible reasons for the highly significant explanatory role of fatigue could be its covering of important functional aspects which may have bearing on work ability. For example, fatigue has both cognitive and physical implications on the functioning of individuals in turn affecting work ability.^35^

The focus on WAS/WAI, so far infrequently used in MS, is one of the strengths of the present study. The use of a large nationwide survey data where the view of PwMS on their work life is studied is another important strength. The linkage of the survey data to other nationwide registers on sociodemographic and clinical data of the PwMS also added to its richness in addressing broader factors.

As to limitations, given the present study was of cross-sectional design, the direction and temporality of the associations found could not be fully elucidated. Longitudinal data could ameliorate this limitation. The difference between respondents and nonrespondents could be a further limitation. In addition, in subgroups with fewer PwMS (i.e., primary and secondary progressive MS and higher EDSS score) lower statistical power was noted.

The findings of the present study add important information on the level of work ability among PwMS using a relatively simple but important measure, WAS, which has so far been rarely studied; and when studied, done with much smaller sample sizes. The findings on the factors associated with WAS are in line with studies where work ability was assessed using measures such as unemployment, sickness absence, disability pension, and income from work.^2,28^ This shows that it can be applied in future studies with relative ease and lower burden to respondents. Although more studies with longitudinal designs would be important to assess the reproducibility of the findings, the current results indicate the potential benefit of the WAS measure.

## Conclusion

The present study documented the level of work ability among PwMS in Sweden where mean WAS was found to be lower than that of the general population. Level of disability (EDSS score), fatigue, and the type of occupation were found to be strong predictors of WAS among other variables. The results suggest that interventions on pervasive symptoms like fatigue could have positive implication on improving work ability considering the strong association it has with work ability. Building on the present findings, further longitudinal studies and data on additional variables such as disease activity could provide stronger evidence on the association of the different sociodemographic, clinical, and self-reported health variables with work ability.

## Supplemental Material

sj-docx-1-mso-10.1177_20552173241304324 - Supplemental material for Factors associated with self-reported work ability among people with multiple sclerosis in Sweden Supplemental material, sj-docx-1-mso-10.1177_20552173241304324 for Factors associated with self-reported work ability among people with multiple sclerosis in Sweden by Fitsum Sebsibe Teni, Alejandra Machado, Jessica Dervish, Katharina Fink, Hanna Gyllensten and Emilie Friberg in Multiple Sclerosis Journal – Experimental, Translational and Clinical
