# Supplementary material for: Factors associated with self-reported work ability among people with multiple sclerosis in Sweden
Source: Mult Scler J Exp Transl Clin. 2025 Jan 7;11(1):20552173241304324. doi: 10.1177/20552173241304324 (PMC11705311; doi:10.1177/20552173241304324)
Supplement: sj-docx-1-mso-10.1177_20552173241304324 - Supplemental material for Factors associated with self-reported work ability among people with multiple sclerosis in Sweden [file sj-docx-1-mso-10.1177_20552173241304324.docx]

# **Supplementary materials**

**Additional information**

**Methods**

**Occupation**

Data on the current occupation of people with multiple sclerosis (PwMS) in the survey was collected using the question ‘what is your current occupation?’ with a direction ‘write in as much detail as possible the occupation that is your main occupation (e.g. an assistant nurse at a nursing home, a journalist at a daily newspaper, a nanny at a preschool, HR consultant at a municipal personnel department).’ The answers were categorized using the Swedish Standard Classification of Occupations (SSYK 2012) [1]. This was recategorized into three groups: managerial, mainly office, and manual occupations as in our another recent study [2].

**Health-related quality of life**

The data on health-related quality of life was collected using the EQ-5D questionnaire. The EQ-5D contains five questions about difficulties in the mobility, self-care, usual activities, pain/discomfort, and anxiety/depression dimensions. In the present study, the three-severity level version of the EQ-5D (EQ-5D-3L) was employed; namely ‘no problems’, ‘some/moderate problems’ and ‘severe/extreme problems’ reported for each dimension [3]. The reported problems were summarized into a single value using the Swedish experience-based EQ-5D-3L value set [4]. Health-related quality of life was grouped into lower and higher than median EQ-5D-3L for further analyses.

**Categorization of DMT use**

The disease-modifying therapies the respondents were using in the two-years leading up to the survey were categorized as high-efficacy and non-high-efficacy DMTs. The high-efficacy DMTs were alemtuzumab, hematopoietic stem cell transplantation, natalizumab, ocrelizumab, ofatumumab and rituximab. The non-high-efficacy DMTs included cladribine, dimethyl fumarate, fingolimod, glatiramer acetate, interferons (interferon beta-1a, interferon beta-1b and peginterferon beta-1a), siponimod and teriflunomide. The categorization was performed based on literature reviews [5],[6], systematic reviews [7],[8], and expert opinions from neurologists in the project.

**Additional long-term disease/condition**

Data on presence of additional long-term disease/condition to multiple sclerosis was collected using the question ‘In addition to multiple sclerosis, do you have any other long-term illness, diagnosis or disability?’. Accordingly, the presence or not of an additional condition was used in the analysis to take comorbidity into consideration.

**Results**

**Interaction effects**

Analysis of the interaction effects between EDSS score and DMT use showed that EDSS score between 3 and 5.5 showed significant interaction with the group with fatigue T score of higher than 50. However, the increase in explained variance in comparison to final adjusted model was very small (0.20%). In contrast, no significant interaction effect was found between EDSS and DMT use categories.

**Subgroup analysis on factors associated with work ability score**

Further analyses in the EDSS score subgroups of 0, 1-2.5, 3-5.5 and 6-9.5 showed a largely similar factors across the four subgroup regression models on factors associated with work ability score. The variables sex, age, education, occupation, presence of an additional long-term disease/condition and fatigue were significant predictors of work ability score in all or most of the models. However, DMT use showed significant association with WAS among PwMS with no or mild disability while no significant association was shown in PwMS with moderate to severe disability. In addition, although not significant among PwMS with no or mild disability, time from treatment start was a significant predictor among PwMS with moderate to severe disability but with very small estimate. Furthermore, r squared statistic showed that the model explained higher proportion of variance in WAS among those with moderate to severe disability than among those with no or mild disability.

# **Table S1. Selection of study population**

| **Variable** | **n** |
| --- | --- |
| PwMS invited to participate in the survey | 8458 |
| PwMS who responded to the survey | 4412 |
| PwMS with data on disease-modifying therapy use | 4226 |
| PwMS with data on type of MS | 4184 |
| PwMS with data on fatigue T score | 4170 |
| PwMS with data on education | 4144 |
| PwMS with data on presence of long-term illness beside MS | 4103 |
| PwMS included in the final analysis | **4103** |

**PwMS**: people with multiple sclerosis; **MS**: multiple sclerosis

**References**

1. **Statistics Sweden**. Meddelanden i samordningsfrågor för Sveriges officiella statistik. SSYK 2012 Standard för svensk yrkesklassificering (Reports on Statistical Co-ordination for the Official Statistics of Sweden. Swedish Standard Classification of Occupations 2012). 2012. Available at: https://www.scb.se/contentassets/c9d055b6f2114b62bd23c33602b56da5/ov9999_2012a01_br_x70br1201.pdf.

2. **Machado A**, **Murley C**, **Dervish J**, **Teni FS**, **Friberg E**. Work Adjustments by Types of Occupations Amongst People with Multiple Sclerosis: A Survey Study. *J Occup Rehabil*. 2023. Available at: https://doi.org/10.1007/s10926-023-10142-2 [Accessed December 20, 2023].

3. **Devlin N**, **Parkin D**, **Janssen B**. An Introduction to EQ-5D Instruments and Their Applications. In: Devlin N, Parkin D, Janssen B, eds. *Methods for Analysing and Reporting EQ-5D Data*. Cham: Springer International Publishing; 2020:1–22. Available at: https://doi.org/10.1007/978-3-030-47622-9_1 [Accessed June 20, 2022].

4. **Burström K, Sun S, Gerdtham U-G, *et al.*** Swedish experience-based value sets for EQ-5D health states. *Qual Life Res*. 2014; **23**(2):431–442.

5. **Giovannoni G**. Disease-modifying treatments for early and advanced multiple sclerosis: a new treatment paradigm. *Current Opinion in Neurology*. 2018; **31**(3):233–243.

6. **Hauser SL**, **Cree BAC**. Treatment of Multiple Sclerosis: A Review. *The American Journal of Medicine*. 2020; **133**(12):1380-1390.e2.

7. **Li H**, **Hu F**, **Zhang Y**, **Li K**. Comparative efficacy and acceptability of disease-modifying therapies in patients with relapsing–remitting multiple sclerosis: a systematic review and network meta-analysis. *J Neurol*. 2020; **267**(12):3489–3498.

8. **Samjoo IA, Worthington E, Drudge C, *et al.*** Efficacy classification of modern therapies in multiple sclerosis. *J Comp Eff Res*. 2021; **10**(6):495–507.
